# The Role of Excitability and Network Structure in the Emergence of Focal and Generalized Seizures

**DOI:** 10.3389/fneur.2020.00074

**Published:** 2020-02-11

**Authors:** Marinho A. Lopes, Leandro Junges, Wessel Woldman, Marc Goodfellow, John R. Terry

**Affiliations:** ^1^Living Systems Institute, University of Exeter, Exeter, United Kingdom; ^2^Wellcome Trust Centre for Biomedical Modelling and Analysis, University of Exeter, Exeter, United Kingdom; ^3^EPSRC Centre for Predictive Modelling in Healthcare, University of Exeter, Exeter, United Kingdom; ^4^Department of Engineering Mathematics, University of Bristol, Bristol, United Kingdom; ^5^Cardiff University Brain Research Imaging Centre, School of Psychology, Cardiff University, Cardiff, United Kingdom; ^6^Centre for Systems Modelling and Quantitative Biomedicine, University of Birmingham, Birmingham, United Kingdom; ^7^Institute for Metabolism and Systems Research, University of Birmingham, Birmingham, United Kingdom

**Keywords:** focal seizures, generalized seizures, neural mass model, ictogenic network, network structure, excitability

## Abstract

Epileptic seizures are generally classified as either focal or generalized. It had been traditionally assumed that focal seizures imply localized brain abnormalities, whereas generalized seizures involve widespread brain pathologies. However, recent evidence suggests that large-scale brain networks are involved in the generation of focal seizures, and generalized seizures can originate in localized brain regions. Herein we study how network structure and tissue heterogeneities underpin the emergence of focal and widespread seizure dynamics. Mathematical modeling of seizure emergence in brain networks enables the clarification of the characteristics responsible for focal and generalized seizures. We consider neural mass network dynamics of seizure generation in exemplar synthetic networks and we measure the variance in ictogenicity across the network. Ictogenicity is defined as the involvement of network nodes in seizure activity, and its variance is used to quantify whether seizure patterns are focal or widespread across the network. We address both the influence of network structure and different excitability distributions across the network on the ictogenic variance. We find that this variance depends on both network structure and excitability distribution. High variance, i.e., localized seizure activity, is observed in networks highly heterogeneous with regard to the distribution of connections or excitabilities. However, networks that are both heterogeneous in their structure and excitability can underlie the emergence of generalized seizures, depending on the interplay between structure and excitability. Thus, our results imply that the emergence of focal and generalized seizures is underpinned by an interplay between network structure and excitability distribution.

## Introduction

Seizures are the hallmark of epilepsy. They are transient events of highly synchronous neuronal activity (1). According to the International League Against Epilepsy, seizures can be classified as focal or generalized, depending on whether one or the two hemispheres are involved at the initial manifestations of seizure activity (1). Seizures may also be classified as unknown onset, when the available information is insufficient to decide whether they are focal or generalized. The classification of seizures precedes the diagnosis of epilepsy type (2), which in turn determines the first line of treatment and respective prognosis (3).

Focal and generalized seizures exhibit distinct electroencephalographic features (1). Whilst generalized seizures are usually associated to generalized spike-wave discharges and polyspike-and-wave complexes (4), focal seizures may emerge in a number of different localized EEG patterns, such as rhythmic spikes, sinusoidal discharges, fast discharges, sharp activity, and background flattening (5). Traditionally, it had been assumed that focal seizures result from localized abnormalities, whilst generalized seizures are the consequence of more widespread pathologies (6). In fact, brain structural abnormalities observable in MRI are a key feature to identify the epileptogenic zone in people considered for epilepsy surgery (7). On the other hand, generalized epilepsies have been associated to widespread structural changes (8). However, recent evidence shows that focal pathologies can be underpinned by widespread and even bilateral phenomena (9, 10), whereas localized foci can drive generalized seizures in rat models (11, 12). It has also been shown that generalized spike wave discharges can be preceded by an increase in neuronal activity in the thalamus and regional decreases in the cortex (13, 14). In the case of refractory epilepsies, the possible involvement of widespread networks in the generation of focal seizures may explain why surgery is often unsuccessful (15, 16).

To address the role of networks in the generation of seizures, a mathematical framework has been proposed (17). Here we extend this mathematical framework to understand the role of network structure and excitability within brain regions across the network on the emergence of focal and widespread seizure activity. In this work we address (i) whether focal seizures are underpinned by heterogeneities in local excitability; (ii) whether generalized seizures are supported by specific network structures that promote global network communication; and (iii) how excitability and network structure interact to give rise to emergent seizure patterns.”

## Materials and Methods

### Mathematical Model

To understand what underpins the emergence of focal and widespread seizure activity in brain networks, we consider a phenomenological model of seizure dynamics, the theta model (18, 19), and a collection of exemplar synthetic networks. In these networks, nodes represent brain regions capable of generating seizure activity, and edges correspond to white matter fibers connecting the regions. In the model, each node is described by a phase which can either fluctuate close to a fixed stable phase or oscillate. These states represent normal and seizure activity, respectively. The transitions between them are driven both by noise and dynamical interactions across the network (see the Supplementary Material for a detailed description of the model). An important parameter in this model is node excitability, which determines how likely a node is in isolation to transit from the stable phase to oscillations. In other words, it defines how close a phase oscillator is to the transition point. This simple model has been shown to be a computationally efficient and reliable approximation of a more complex and biophysical realistic model of epileptiform dynamics (18). Here we use it to understand how network structure and tissue heterogeneities determine how focal or widespread seizures are.

### Ictogenic Variance

To quantify network dynamics, we use the concept of Brain Network Ictogenicity (BNI) (17–21). The BNI represents the propensity of a network to generate seizures and is computed as the average time that each node spends in seizure activity (see the Supplementary Material for a detailed description of BNI). This quantity depends on a global scaling of the coupling, via the parameter K. As K is increased, interactions between nodes become stronger and consequently the network becomes more likely to seize (BNI increases) (18). Different nodes are characterized by different curves of BNI as a function of K, where the most ictogenic nodes are those for which BNI is larger at all values of K. In order to account for these changes in BNI as a result of changes in coupling strength, we calculate the quantity $\hat{BNI}$, which is the integral of BNI as a function of K (see the Supplementary Material).

To distinguish whether a network generates focal or generalized seizure activity, we introduce the Ictogenic Variance (IV):

$$\begin{array}{l} IV=Var\left(\hat{\text{BNI}}\right) \end{array}$$

**Figure 1:**
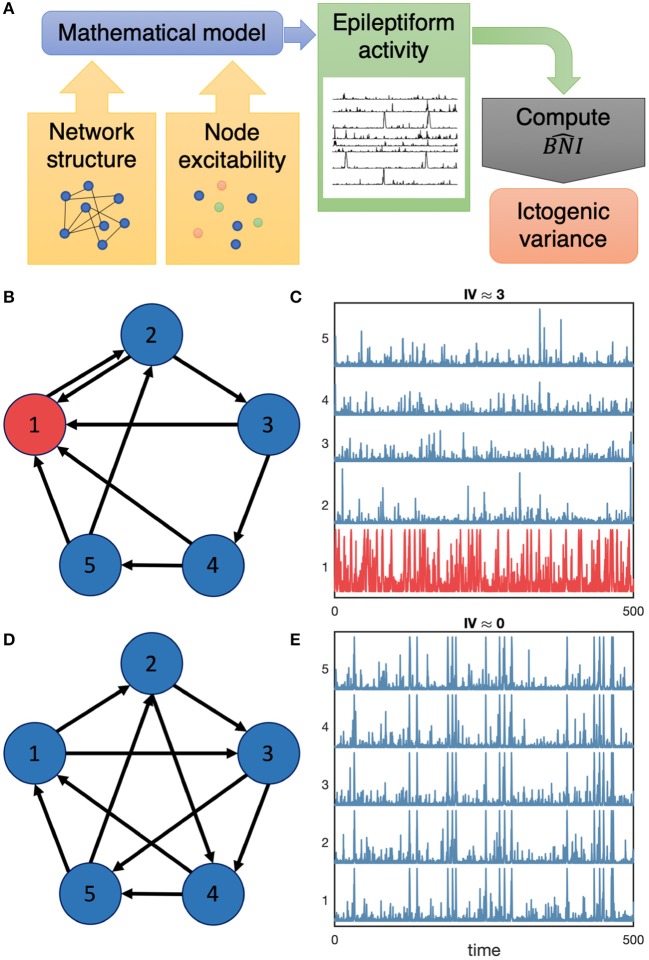
Flowchart of our methodology and illustrative examples of focal and widespread seizure activity. Panel **(A)** displays a schematic summary of our methods: we use a mathematical model to understand the role of network structure and node excitability on the emergence of focal and generalized seizure activity by means of computing the $\hat{BNI}$ and subsequently the ictogenic variance. We consider synthetic networks such as the networks represented in **(B,D)**. We then place a model of seizure transitions onto the nodes of the networks and compute the emerging dynamics in the networks. **(C,E)** show model generated activity in the networks **(B,D)**, respectively. High amplitude spike activity represents epileptiform activity in this model. In panel **(C)**, node 1 produces a higher rate of spike activity compared to other nodes, whereas in **(E)** all nodes generate similar activity. Consequently, the Ictogenic Variance (IV) is higher in network **(B)**, *IV* ≈ 3, compared to network **(D)**, *IV* ≈ 0.

IV is the variance of the $\hat{BNI}$ across network nodes ($\hat{\text{BNI}}=\left\{\hat{BNI_{1}},\hat{BNI_{2}},\ldots ,\hat{BNI_{N}}\right\}$, where $\hat{BNI_{i}}$ is the $\hat{BNI}$ of node *i*, and *N* is the number of nodes in the network). Low IV values imply that most nodes have similar ictogenicities, whereas high IV values mean that nodes differ widely with respect to ictogenicity. Low IV is thus interpreted as indicative of a network that supports generalized activity, whereas high IV indicates the existence of foci. This definition of IV further allows us to study the mechanisms of seizure emergence as belonging to a spectrum spanning from focal to generalized, rather than classifying seizures binarily as generalized or focal. Figure 1 illustrates our framework.

### Network Topologies and Heterogeneous Excitabilities

To understand what underlies the emergence of more focal or generalized seizure patterns across networks, we compute the IV for a variety of different network topologies. We consider regular, small-world, random, and scale-free networks, both directed and undirected consisting of 64 nodes (see the Supplementary Material for more details about the construction of networks and parameters used). We focused on these network topologies in order to study limiting cases with regards to key network properties. We studied 1,010 networks in total (see Supplementary Table S1). Furthermore, we considered both networks with homogeneous and heterogeneous excitabilities. Node excitabilities define how likely a node is to generate seizures in isolation. Hence, when all node excitabilities are the same (homogeneous), node ictogenicity is exclusively a function of network structure; whereas for heterogeneous excitabilities, ictogenicity across the network is determined by both network topology, and excitability distribution. We consider two heterogeneous excitability distributions apart from a homogeneous excitability distribution. In the first case, we introduce a small fraction of hyper-excitable nodes in the network (i.e., nodes with higher excitabilities compared to others). Our aim was to understand whether the presence of these hyper-excitable nodes could increase the IV of the networks. In the second case we consider node excitabilities proportional to the inverse of the node degree (see implementation details in the Supplementary Material). In this case, we aimed to test whether a heterogeneous excitability distribution could balance the effect of network degree heterogeneity. According to our previous findings (18), we expect that nodes with high number of connections are the most ictogenic (higher $\hat{BNI_{i}}$), and therefore we posed the question as to whether by decreasing their excitability the IV across the network would decrease.

## Results

To understand how network structure and excitability distributions determine whether emerging seizure dynamics are widespread or focal, we measured the ictogenic variance in a variety of network topologies with both homogeneous and heterogeneous excitabilities across network nodes.

**Figure 2:**
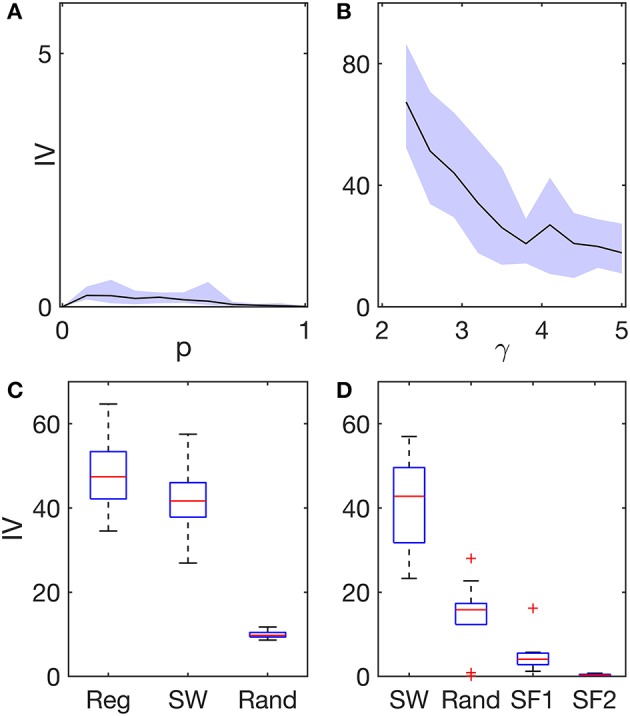
Ictogenic variance (IV) in different network structures and excitability distributions. **(A)** IV as a function of the network re-wiring probability *p* (Watts-Strogatz algorithm). At *p* = 0 the network is regular, whereas at *p* = 1 the network is random. In between, 0 < *p* < 1, the networks are small-world. **(B)** IV as a function of the exponent γ (static model). The exponent γ characterizes the heterogeneity of a scale-free network with regards to node degree: lower γ corresponds to higher degree heterogeneity. In both **(A,B)**, excitabilities across network nodes were homogeneous. Black lines represent the average IV across 10 network realizations per network topology, and the shaded areas represent the maximum variability across these network realizations. **(C)** IV in regular (Reg), small-world (SW, *p* = 0.1), and random (Rand) networks with heterogeneous excitability distributions. In these networks, a small fraction of nodes (~9%) was selected at random and defined as hyper-excitable. **(D)** IV in small-world, random, and scale-free networks (SF1: γ = 2.3, and SF2: γ = 5). In these networks, node excitability was defined as inversely proportional to node degree. The boxplots in **(C,D)** correspond to different runs over 10 network realizations per network topology. The boxes in **(C)** further consider five different runs per network realization using different random assignments of hyper-excitable nodes. All networks were undirected and consisted of 64 nodes and had a mean degree *c* = 4. See the Supplementary Material for a detailed description of how the networks were constructed and the excitability distributions implemented.

First, we focused on networks with homogeneous excitability distributions, i.e., where all network nodes are equivalent apart from network topological properties. We used two different algorithms to generate network topologies: the Watts-Strogatz algorithm (22) to obtain regular, small-world, and random networks; and the static model (23) to generate scale-free networks. Figure 2A shows the IV in regular (*p* = 0), small-world [0 < *p* < 1], and random (*p* = 1) networks. We observe that all IV values are close to zero, irrespective of topology, meaning that emerging seizure patterns are widespread in these networks. We note, however, that regular and random networks are characterized by smaller IV values than small-world networks. We interpret this as a consequence of regular networks having all nodes equivalent and in random networks heterogeneity being low, therefore activity is more generalized. Note that if two nodes are equivalent, then their propensity to be recruited into seizure dynamics is the same. In small-world networks, IV values are slightly higher, showing that heterogeneities in node degree (number of connections) and mean distance between nodes across the network enable some nodes to have higher ictogenicity than others, thus increasing the overall IV.

Figure 2B shows IV values in scale-free networks characterized by degree distributions *P*(*k*) ∝ *k*^−γ^. IV values in Figure 2B are much larger than those observed in regular, small-world, and random networks in Figure 2A, despite the excitability distribution being homogeneous in all these networks. Furthermore, we find that the smaller the degree distribution exponent γ is, the higher the network IV is. Note that the smaller the exponent is, the more heterogeneous the network is with respect to node degree (24). Thus, we find that the more heterogenous a network is with regards to node degree, the more likely it is to generate more focal seizure dynamics.

We then considered two different excitability distributions across network topologies: (i) six randomly selected nodes were defined as “hyper-excitable;” and (ii) node excitabilities were defined as inversely proportional to node degree, such that nodes with high degree would have low excitability (see the Supplementary Material for more details and motivation). Figure 2C shows that the presence of a small collection of hyper-excitable nodes increases the IV significantly in regular, and small-world networks, but less so in random networks (compare with Figure 2A). Given that in regular networks, nodes are all equivalent apart from the hyper-excitable nodes, it is clear that the hyper-excitable nodes define foci. The IV of small-world networks is similar to that of regular networks, but takes slightly lower values, presumably due to the existence of long-range connections which allow activity to propagate throughout the network. In contrast, hyper-excitable nodes do not have such a strong impact in random networks, where the IV is clearly lower than in regular and small-world networks. The main difference between random and small-world networks is that random networks have low clustering coefficients (22). These results thus suggest that high clustering promotes focal activity.

Figure 2D demonstrates the effect of setting node excitabilities to values that are inversely proportional to node degree. We find that in this case, small-world and random networks display much higher IV values compared to scale-free networks. Note that for homogeneous excitability distributions, we observed much higher IV in scale-free networks compared with the other networks (see Figures 2A,B), whereas when excitability is inversely proportional to node degree, we find the opposite (Figure 2D). Whilst in scale-free networks with homogeneous excitabilities, nodes with high degree are more likely to seize compared to other nodes, when excitabilities are inversely proportional to node degree, low excitability prevents highly connected nodes from being the focus of seizure activity. Interestingly, whilst this choice of excitability distribution reduces the IV in scale-free networks, it increases the IV in both small-world and random networks. This suggests that excitability may compensate the role of degree heterogeneity in the generation of focal activity in scale-free networks, whereas in small-world and random networks it is responsible for promoting focal activity in networks that would otherwise support widespread seizure activity. Consequently, these results show that the emergence of generalized and focal seizure dynamics may only be understood if both network structure and excitability distribution are taken into account.

## Discussion

In this study we examined how network structure and the distribution of excitability throughout a network underpin the emergence of widespread and localized seizure activity. We explored regular, small-world, random, and scale-free networks with homogeneous excitability distributions, and observed that networks with more regular topologies, i.e., having structures for which nodes are very similar, support generalized seizures, whereas more heterogeneous networks, where nodes may have significantly different number of connections, underlie focal activity (see Figures 2A,B). Heterogenous excitability distributions in networks that were otherwise homogeneous (e.g., regular networks) also enabled the emergence of focal seizure patterns (see Figure 2C). We further showed that heterogeneous node excitability may reduce the ictogenic variance, i.e., make seizure activity more widespread in networks that supported otherwise more focal activity (compare IV values of scale-free networks in Figures 2B,D). This shows that whether seizure activity is more focal or widespread is determined by a complex interplay between brain network structure and tissue heterogeneities (here represented as different excitabilities across the network). In particular, heterogeneity in either intrinsic excitability or connectivity of nodes is a necessary but not sufficient condition for the emergence of focal seizure activity. In other words, our results suggest that focal seizures are either due to heterogeneity in network topology or due to a localized “focus.” However, the existence of these heterogeneities does not imply the emergence of focal activity, because the two types of heterogeneity may balance each other. On the other hand, homogeneous network topologies may support generalized seizure activity provided that excitabilities are sufficiently homogeneous across the network. Together, these findings may help explain evidence showing that focal pathologies can be underpinned by widespread phenomena (9, 10), and localized foci can be responsible for generalized seizures in rat models (11, 12).

Our findings may also be used to interpret the functioning of healthy brain networks. Namely, healthy human connectomes have been found to have rich-club structure (25), which is a network structure highly heterogeneous with regard to the distribution of connections. In these networks, it is conceivable that connectivity and tissue excitability may balance each other to sustain healthy brain activity.

## Limitations

The framework used in this study has a few limitations. First, the considered networks were abstract, and we did not account for the spatial location of nodes. A node in our networks represented a small brain region that is capable of generating seizure activity without further concerns to the actual physiology and anatomy of such brain tissue. Likewise, connections described ways activity could propagate between nodes, and were therefore also phenomenological in nature. Furthermore, by not taking into account the physical location of nodes, the framework could not describe whether nodes involved in seizure activity were spatially close or distant from each other (e.g. in different brain hemispheres). These choices meant that on one hand we could not study spatial features of seizures, and on the other hand we could not explore the potential physiological mechanisms responsible for the generation of focal and generalized seizures. Instead, we focused our analysis on the variability in their involvement in seizure activity across networks. Thus, in this study focal activity was inferred from the existence of network nodes with dissimilar activities compared to the average activity of other nodes. Whilst this measure of ictogenic variance quantifies temporal patterns of network dynamics, it does not take into account different activity patterns across nodes and specific correlations therein. Furthermore, the framework does not account for seizure onset and propagation (e.g., seizures with secondary generalization). More sophisticated models would need to be considered to describe these phenomena (26). Also, we only considered a finite number of artificial network topologies (regular, small-world, random, and scale-free networks), rather than studying real brain network topologies. The reason to focus on such networks was to study limiting cases in order to build understanding (27). Finally, our model is phenomenological, and thus does not include physiological details of the brain. Instead, it describes fundamental mechanistic principles that capture emergent dynamical phenomena (18). In particular, the model assumes that the transition to seizures is driven by noise and network activity and is described by a specific bifurcation (see the Supplementary Material for details). However, other mechanisms are possible (28–30). For example, it has been suggested that bistability may underlie the occurrence of generalized absence seizures (28), whereas focal seizures have been modeled as the consequence of a slowly changing parameter (26). Thus, it is necessary to explore whether our findings are model-dependent (31). Future studies may therefore address these shortcomings to clarify the pathophysiological mechanisms that underlie the emergence of focal and generalized seizures. Crucially, our results indicate that both excitability distribution and network structure should be taken into account in such detailed studies.

## Conclusions

In recent years, a substantial literature has focused on trying to understand epilepsy as either a network change (10, 32), or an imbalance between excitation and inhibition at the microscale level (33). Our findings suggest that to understand the mechanisms of seizure emergence, and to develop diagnostic tools of epilepsy type, it may be necessary to consider together network changes and dynamic imbalances between excitation and inhibition within nodes in the network. Heterogeneities in network structure or local excitabilities may independently underlie the emergence of focal seizures. However, the simultaneous occurrence of both types of heterogeneity may constitute a balanced regime from which generalized seizures can emerge. Future research should aim to disentangle network structure from network node excitabilities and find methods to measure the importance of structure relative to tissue heterogeneities in emergent seizure dynamics.

## Data Availability Statement

The code and synthetic networks generated are available upon request.

## Author Contributions

ML, MG, and JT: study concept and design, results interpretation, and manuscript drafting and revision. ML: formal analysis. LJ and WW: manuscript revision.

### Conflict of Interest

JT and WW are co-founders of Neuronostics. The remaining authors declare that the research was conducted in the absence of any commercial or financial relationships that could be construed as a potential conflict of interest.
