# Supplementary material for: The Role of Excitability and Network Structure in the Emergence of Focal and Generalized Seizures
Source: Front Neurol. 2020 Feb 11;11:74. doi: 10.3389/fneur.2020.00074 (PMC7027568; doi:10.3389/fneur.2020.00074)
Supplement: Supplementary file 1 [file Data_Sheet_1.PDF]

Supplementary Material

1 Supplementary Methods

1.1 Mathematical model

The theta model comprises a single ordinary differential equation (ODE):

$$\dot{\theta}_j = \left(1 - \cos(\theta_j)\right) + \left(1 + \cos(\theta_j)\right) I_j(t),$$

where θ_j is the phase of an oscillator j, and $I_j(t)$ its time-dependent input current. This is the canonical form of the saddle-node on invariant circle (SNIC) bifurcation. At $I_j < 0$ the phase oscillator is at rest (stable fixed point), whereas at $I_j > 0$ it is oscillating (a stable limit cycle). The SNIC bifurcation occurs at $I_j = 0$. Although this model was initially proposed to represent the activity of single neurons (1), here it represents the activity of neural masses (2). In this phenomenological model, the neural mass may transit between a "normal state" when its phase is at rest, and a "seizure state" when its phase is oscillating.

The input current I_j includes the neural mass's excitability ($I_0^{(j)}$), noise ($\xi^{(j)}(t)$), and interaction with other neural masses:

$$I_j(t) = I_0^{(j)} + \xi^{(j)}(t) + \frac{K}{N} \sum_{i \neq j} a_{ij} [1 - \cos(\theta_i - \theta_i^{(s)})]$$

where K is a global scaling coupling that scales the interaction with other phase oscillators, N is the number of oscillators (nodes) in the network, a is the adjacency matrix that describes the network topology (a_{ij} is either 0 or 1 depending on whether there is a connection from node i to node j), $1 - \cos(\theta_i - \theta_i^{(s)})$ is the output of the in-neighbour i, and $\theta_i^{(s)}$ is its steady state (the output is zero if the oscillator is resting, and reaches a maximum at $\theta_i = \theta_i^{(s)} + \pi$). The uncoupled steady state $\theta_i^{(s)}$ is given by

$$\theta_i^{(s)} = -\mathrm{Re}\left\{\cos^{-1}\left(\frac{1+I_0^{(i)}}{1-I_0^{(i)}}\right)\right\}.$$

We considered the excitability $I_0^{(j)} < 0$, so that the oscillators would be resting in isolation. More excitable phase oscillators were set with larger $I_0^{(j)}$, i.e. closer to zero, as described below. Every oscillator was excited by Gaussian noise with zero mean and standard deviation $\sigma = 0.6$ as in previous studies (2,3).

1.2 Brain Network Ictogenicity and Ictogenic Variance

We quantify the propensity of a network to generate seizure activity using the concept of *Brain Network Ictogenicity* (BNI):

$$BNI_i(K) = \frac{t_{sz}^{(i)}(K)}{T}$$

where $t_{sz}^{(i)}$ is the time that node i spends in the seizure state during a total simulation time T (we use $T = 4 \times 10^6$ time steps and the seizure state was defined as any activity above a threshold as defined in (2)). To avoid an arbitrary choice of K (3), we calculate

$$\widehat{BNI}_\mathrm{i} = \int_{K_1}^{K_2} BNI_i(K)\, dK$$

The interval $[K_1, K_2]$ was chosen for each network such that the argument could vary from 0 to 1 for all nodes, i.e. from all oscillators at rest to the whole network oscillating. This has been shown to be a robust redefinition of BNI (3). We then defined the *Ictogenic Variance* (IV) as

$$\mathrm{IV} = \frac{1}{N}\sum_{i=1}^{N}\left(\widehat{BNI_\iota} - \langle\widehat{BNI}\rangle\right)^2$$

where $\langle\widehat{BNI}\rangle$ is the average $\widehat{BNI_\iota}$ across the network.

1.3 Network topologies

To study the role of network structure on the IV, we considered phase oscillators in regular, small-world, random, and scale-free networks, both directed and undirected (4-9). These networks represent limiting cases with regards to key network properties, namely with respect to degree distributions, clustering coefficient, and average path length. Whereas regular, small-world, and random networks are network topologies where degree variability is low, in scale-free networks there are nodes highly connected (hubs). Furthermore, clustering coefficients are high in regular and small-world networks, but not in random and scale-free networks. Finally, average path length is relatively high in regular networks compared to the other considered network topologies. To construct a set of small-world networks we used the Watts-Strogatz algorithm (4) and 9 rewiring probabilities ($p = 0.1, 0.2, \ldots, 0.9$). Note that for $p = 0$ we obtain regular networks, whereas for $p = 1$ the algorithm generates random networks (4). We used the static model (5) to generate undirected scale-free networks and the Albert-Barabási algorithm (6) to construct directed scale-free networks. In the case of undirected scale-free networks, we studied 11 different topologies characterized by degree distributions $P_k \propto k^{-\alpha}$ with a range of exponents $\alpha = 2, 2.3, 2.6, \ldots, 5$. Note that the smaller the exponent α is, the more heterogeneous the network is with respect to the number of connections per node. All networks consisted of $N = 64$ nodes and for each network topology we considered three mean degrees $c = 4, 8$, and 16. We discarded networks with disconnected components and considered 10 network realizations per network topology. In total we studied 1010 networks (see Supplementary Table 1).

1.4 Heterogeneous excitability distributions

We considered both networks where all nodes have the same excitabilities ($I_0 = -1.2$), and networks where excitabilities differ between nodes. In the case of heterogeneous excitabilities, we studied two different types of distributions. In the first case, we defined a small fraction of nodes as hyper-excitable, i.e. nodes with higher excitability than the other nodes with fixed excitability ($I_0 = -1.2$). We selected six nodes at random (i.e. about 9% of the network) and distinguished them with higher excitabilities

($I_0^{(h)} > I_0$). We studied their impact on network dynamics in regular, small-world, and random networks. For each network realization, we considered 5 different random selections of hyper-excitable nodes. We performed simulations with $I_0^{(h)} = -1$ and -0.1, i.e. with "weak" and "strong" hyper-excitable nodes.

Additionally, we considered excitabilities inversely proportional to the node's number of connections (degree). In the directed networks, we considered $I_0^{(i)}$ proportional to both node in- and out-degree separately. We fixed the range of excitabilities, such that the node with the largest number of connections had the minimum excitability $I_0^{(i)}$, whist the node with the smallest number of connections had the maximum $I_0^{(i)}$. We studied five intervals of I_0: $[-2.5, -0.5]$, $[-2.5, -1.5]$, $[-2, -1]$, $[-1.5, -0.5]$, and $[-5, -0.5]$.

2 Supplementary Tables

	**Networks**	
**Topologies**	**Undirected**	**Directed**
**Regular**	10 n.r.	10 n.r.
**Small-world**	$p = 0.1, 0.2, \dots, 0.9$<br>10 n.r. per p	$p = 0.1, 0.2, \dots, 0.9$<br>10 n.r. per p
**Random**	10 n.r.	10 n.r.
**Scale-free**	$\alpha = 2, 2.3, 2.6, \dots, 5$<br>10 n.r. per α	$\alpha = 3$<br>10 n.r.

Supplementary Table S1: Summary of all networks considered. p is the rewiring probability to obtain small-world networks, and α is the degree distribution exponent of scale-free networks. We considered 10 network realizations (n.r.) per topology. The table thus represents 34 topologies and a total of 340 networks. We generated networks with three different mean degrees, $c = 4, 8, 16$ (in the case of directed networks, it corresponds to mean in- and out- degree). We did not consider scale-free networks with $\alpha = 2$ and $c = 4$, as these did not meet the condition of no disconnected components. Therefore, we studied $340 \times 3 - 10 = 1010$ networks.

3 Supplementary References

(1) Ermentrout GB, Kopell N. Parabolic Bursting in an Excitable System Coupled with a Slow Oscillation. *SIAM J Appl Math* (1986) 46: 233–253. doi:10.1137/0146017

(2) Lopes MA, Richardson MP, Abela E, Rummel C, Schindler K, Goodfellow M, et al. An optimal strategy for epilepsy surgery: Disruption of the rich-club? *PLoS Comput Biol* (2017) 13:e1005637. doi:10.1371/journal.pcbi.1005637

(3) Lopes MA, Richardson MP, Abela E, Rummel C, Schindler K, Goodfellow M, et al. Elevated ictal Brain network ictogenicity enables Prediction of Optimal seizure control. *Front Neurol* (2018) 9:98. doi:10.3389/fneur.2018.00098

(4) Watts DJ, Strogatz SH. Collective dynamics of 'small-world' networks. *Nature* (1998) 393:440. doi:10.1038/30918

(5) Goh KI, Kahng B, Kim D. Universal Behavior of Load Distribution in Scale-Free Networks. *Phys Rev Lett* (2001) 87:278701. doi:10.1103/PhysRevLett.87.278701

(6) Albert R, Barabási AL. Topology of evolving networks: local events and universality. *Phys Rev Lett* (2000) 85:5234. doi:10.1103/PhysRevLett.85.5234

(7) Newman MEJ. The Structure and Function of Complex Networks. *Soc Ind Appl Math Rev* (2003) 45:167–256. doi:10.1137/S003614450342480

(8) Lee DS, Goh KI, Kahng B, Kim D. Evolution of scale-free random graphs: Potts model formulation. *Nucl Phys B* (2004) 696:351. doi:10.1016/j.nuclphysb.2004.06.029

(9) Mišić B, Sporns O, McIntosh AR. Communication Efficiency and Congestion of Signal Traffic in Large-Scale Brain Networks. *PLoS Comput Biol* (2014) 10. doi:10.1371/journal.pcbi.1003427
